# The epithelial era of asthma research: knowledge gaps and future direction for patient care

**DOI:** 10.1183/16000617.0221-2024

**Published:** 2024-12-18

**Authors:** Christopher E. Brightling, Gianni Marone, Helena Aegerter, Pascal Chanez, Enrico Heffler, Ian D. Pavord, Klaus F. Rabe, Lena Uller, Del Dorscheid, the Epithelial Science Expert Group

**Affiliations:** 1 Institute for Lung Health, National Institute for Health and Care Research Leicester Biomedical Research Centre, University of Leicester, Leicester, UK; 2 Department of Translational Medical Sciences and Center for Basic and Clinical Immunology Research, School of Medicine, University of Naples Federico II, Naples, Italy; 3 Institute of Experimental Endocrinology and Oncology, National Research Council of Italy, Naples, Italy; 4 Laboratory of Immunoregulation and Mucosal Immunology, VIB-UGent Center for Inflammation Research, Ghent, Belgium; 5 Department of Internal Medicine and Pediatrics, Ghent University, Ghent, Belgium; 6 Department of Respiratory Diseases, Aix-Marseille University, Marseille, France; 7 Department of Biomedical Sciences, Humanitas University, Pieve Emanuele (MI), Italy; 8 Personalized Medicine, Asthma and Allergy, IRCCS Humanitas Research Hospital, Rozzano (MI), Italy; 9 Respiratory Medicine, National Institute for Health and Care Research Oxford Biomedical Research Centre, Nuffield Department of Medicine, University of Oxford, Oxford, UK; 10 LungenClinic Grosshansdorf, Member of the German Center for Lung Research (DZL), Grosshansdorf, Germany; 11 Chirstian-Alrechts University Kiel, Member of the German Center for Lung Research (DZL), Kiel, Germany; 12 Unit of Respiratory Immunopharmacology, Department of Experimental Medical Science, Lund University, Lund, Sweden; 13 Center for Heart Lung Innovation, Department of Medicine, University of British Columbia, Vancouver, BC, Canada; 14 A full list of the Epithelial Science Expert Group members can be found in the Acknowledgements section; 15 Joint first authors

## Abstract

The Epithelial Science Expert Group convened on 18–19 October 2023, in Naples, Italy, to discuss the current understanding of the fundamental role of the airway epithelium in asthma and other respiratory diseases and to explore the future direction of patient care. This review summarises the key concepts and research questions that were raised. As an introduction to the epithelial era of research, the evolution of asthma management throughout the ages was discussed and the role of the epithelium as an immune-functioning organ was elucidated. The role of the bronchial epithelial cells in lower airway diseases beyond severe asthma was considered, as well as the role of the epithelium in upper airway diseases such as chronic rhinosinusitis. The biology and application of biomarkers in patient care was also discussed. The Epithelial Science Expert Group also explored future research needs by identifying the current knowledge and research gaps in asthma management and ranking them by priority. It was identified that there is a need to define and support early assessment of asthma to characterise patients at high risk of severe asthma. Furthermore, a better understanding of asthma progression is required. The development of new treatments and diagnostic tests as well as the identification of new biomarkers will also be required to address the current unmet needs. Finally, an increased understanding of epithelial dysfunction will determine if we can alter disease progression and achieve clinical remission.

## Shareable abstract

**This review introduces the “epithelial era” of asthma research and the vital role of the epithelium in airway diseases. It describes expert-identified knowledge gaps in the current understanding of asthma management and strategies to improve patient care.** https://bit.ly/3NsTNef

## Introduction

**FIGURE 1:**
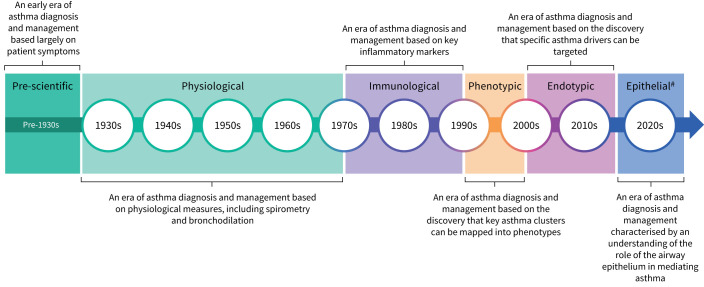
Asthma management through the ages. The time frames noted in this overview are approximate date ranges for each era. ^#^: The epithelial era is based on a current theoretical era of ongoing scientific research focused on the epithelium.

Asthma has been recognised as a disease since antiquity [1]. In the pre-scientific era of asthma research (figure 1), Hippocrates was one of the first physicians who identified the relationship between asthma and environmental triggers [1]. The Greek physician Aretaeus of Cappadocia gave the first accurate description of asthma and noted that symptoms included chest heaviness, difficulty breathing, tiredness and cough [1]. Years of innovative research through the seventeenth and eighteenth centuries culminated in the invention of the spirometer and the coining of the term “vital capacity” by John Hutchinson, an English physician, in 1846 [2]. In the first half of the twentieth century, a distinguished Boston physician named Francis Rackemann was the first to highlight the heterogeneity of asthma [3]. The physiological era of asthma research, beginning in the 1930s, saw the first publication of literature detailing the effectiveness of bronchodilation for patients with severe asthma [2]. In 1956, a report of a UK Medical Research Council trial described the use of systemic corticosteroids for asthma [4]. Importantly, in 1958 it was first reported that sputum eosinophilia may determine the response to oral corticosteroids (OCS) and inhaled corticosteroids (ICS) [5]. In the 1960s, immunoglobulin E (IgE) was identified as the immunoglobulin responsible for IgE sensitisation [6]. In the early 1970s, it was demonstrated that treatment with beclomethasone allowed patients to cease OCS treatment without loss of asthma control, thereby reducing the number of adverse effects associated with asthma treatment [7].

The immunological era of asthma research occurred between 1970 and 1990 (figure 1). In the 1970s, clinical trial data demonstrated that the use of ICS could reduce airway eosinophilic and mast cell inflammation [8]. During this era, the roles of primary effector cells (such as mast cells and basophils) and type-2 (T2) cytokines (such as interleukin (IL)-4, IL-5 and IL-13) in asthma were identified [9, 10]. The hypothesis of early- and late-phase reaction of the immune response to external triggers was also suggested [11]. In the late 1990s, two independent studies suggested that patients with severe asthma could be divided into two distinct subgroups based on the presence or absence of eosinophils [12, 13]. There was increased recognition of the heterogeneity of asthma and, in the early 2000s, asthma phenotypes were first described [14]. Haldar *et al*. [15] first described the classification of clinical phenotypes of asthma using a cluster analysis. Importantly, eosinophils were found to be key effector cells in the pathogenesis of severe exacerbations in asthma and the concept of T2-high and T2-low inflammatory asthma phenotypes was established [16, 17]. Subsequently, a number of targeted agents were investigated as potential treatments, including antibodies that target the IgE, IL-4, IL-5 or IL-13 pathways [18]. More recent data have demonstrated that biomarkers such as fractional exhaled nitric oxide (*F*_ENO_) and sputum eosinophils can be used for the sub-stratification of T2-high airway disease and can assist in therapeutic decision-making [19]. Subsequently, aided by new technologies capable of qualitative and quantitative analysis of immune cells, researchers and healthcare professionals in the immunological era focused on defining and treating asthma primarily as a disease characterised by various aspects of inflammation. However, it is now known that the airway epithelium and other structural components of the lung respond directly to environmental risk factors for asthma and thus are key initiators and orchestrators of the inflammatory cascade [20].

Consequently, the authors of this review suggest that we are in an era of research that is focusing on the fundamental role of the airway epithelium in inflammation in asthma and other respiratory diseases [21]. The airway epithelium is the first line of defence against pathogenic environmental factors. It plays a key role in initiating host defence and controlling immune responses [21] and epithelial dysfunction is integral to the development and progression of asthma [22]. Upstream epithelial cytokines, collectively termed alarmins (such as thymic stromal lymphopoietin (TSLP) and IL-33), have been established as key drivers of asthma pathobiology from the top of the immunologic cascade [23, 24]. Therefore, within this “epithelial era”, the importance of monoclonal antibodies that inhibit the alarmins has been recognised.

The Epithelial Science Expert Group convened on 18–19 October 2023, in Naples, Italy, to define the current understanding of and future research direction for the diagnosis and management of asthma and other respiratory diseases within the emerging epithelial era. This review summarises the key concepts that were raised. As an introduction to the epithelial era of asthma research, the evolution of asthma management throughout the ages was discussed and the role of the epithelium as an immune-functioning organ was elucidated. The role of the epithelium in airway diseases beyond severe asthma was considered, focusing on the role of the epithelium in chronic rhinosinusitis, an upper airway disease. Of note, the biology and application of biomarkers in patient care was also discussed. Workshops were held to identify key research themes in epithelial science that will further support broader patient care goals in respiratory disease. The existing knowledge gaps related to the role of the epithelium in respiratory disease were identified and opportunities to further strengthen knowledge and understanding in this area were discussed.

## The epithelial era of asthma research

### The role of the epithelium as an immune-functioning organ

A high degree of cellular heterogeneity has been observed within the airway epithelium, with each cell type playing a functionally distinct role in health and disease (table 1) [25]. The airway epithelium is regarded as an interface, strategically positioned to function both as a physical barrier and an immune organ [26, 27]. In healthy individuals, its physical barrier plays an important role in the preservation of immune homeostasis in the lung and protection from pathogens and pollutants, which can induce inflammation at the epithelial level [26]. The epithelium also engages in consistent immunological activity, maintaining respiratory health in response to allergen, bacterial or viral exposure [28]. Properties of the epithelium, such as mucociliary clearance, augment an appropriate and balanced host defence response to these airborne insults [29].

**TABLE 1:** The role of airway epithelial cells [25, 28, 137–141]

Cell type	Role
**Ciliated**	Airway homeostasis
	Trapping and expelling microorganisms
	Mucociliary clearance
	Produces TSLP
**Goblet**	Produces mucus
	Mucociliary clearance
**Basal**	Principal stem cells of the airway
	Airway homeostasis
	Epithelial regeneration following injury
	Produces IL-33
**Club**	Stem cells of the airway
	Epithelial repair
	Generate ciliated and goblet cells
**Tuft**	Rare epithelial cells
	Generate cysteinyl leukotrienes
	Produces IL-25
**Pulmonary neuroendocrine**	Communicators between the immune system and nervous system

IL: interleukin; TSLP: thymic stromal lymphopoietin.

**FIGURE 2:**
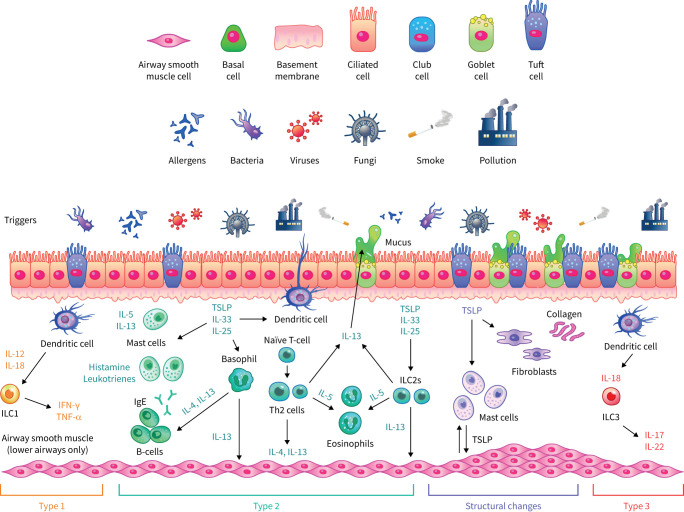
The role of the epithelium in upper and lower airway diseases. The airway epithelium consists largely of three main cell types, namely ciliated cells, secretory (goblet and club) cells and basal cells. Ciliated and secretory cells facilitate clearance of mucus and debris from the upper and lower airways. The upper airways comprise approximately 20% of goblet cells which secrete mucins and other glycoproteins that contribute to mucus viscosity. In the lower airways, there is an increased presence of club cells, which are implicated in epithelial repair and facilitate homeostasis. Similar inflammatory processes (classified as T1, T2 and T3 responses) are associated with both upper and lower airway diseases, in which interaction with external triggers results in the release of cytokines and inflammatory mediators. Structural changes to the airway, termed airway remodelling, may also occur. IFN: interferon; IgE: immunoglobulin E; IL: interleukin; ILC: innate lymphoid cell; Th: T-helper; TNF: tumour necrosis factor; TSLP: thymic stromal lymphopoietin.

In patients with asthma, and particularly severe asthma, exposure to environmental triggers (for example, allergens, microbial products, pollutants or detergents) can dysregulate and damage the airway epithelium [20, 21]. In response to this, the epithelium releases numerous cytokines and growth factors that act on neighbouring cells and the submucosa to induce various features of asthma, including both inflammation and structural changes such as subepithelial fibrosis, inflammatory angiogenesis and remodelling of the airway wall (figure 2) [30]. The cytokines released include alarmins and damage-associated molecular patterns, such as TSLP, IL-33 and IL-1α, which have been shown to play prominent roles in allergic sensitisation and airway inflammation *via* activation of various types of immune cell including dendritic cells, T-helper 2 cells, type 2 innate lymphoid cells, mast cells and eosinophils [23, 24, 30, 31]. Loss of epithelial barrier function may lead to enhanced pro-inflammatory activity and promote a T2-driven immune response through mechanisms that include the loss of expression of cell adhesion molecules, such as E-cadherin, as part of a process known as epithelial-to-mesenchymal transition. It has been hypothesised that airway epithelial loss of E-cadherin is a critical step in asthma pathogenesis [32]; supportive of this, siRNA silencing of E-cadherin resulted in reduced epithelial resistance in bronchial epithelial cells and led to increased expression of TSLP and C–C motif chemokine ligand 17 [22, 33].

Respiratory viruses have also been shown to impair epithelial barrier function by inducing perturbations in the continuity of the epithelial barrier and interfering with tight junctions, leading to an increase of paracellular permeability [34]. Exposure to respiratory viral infections in childhood is an important risk factor in the development of asthma and is the principal cause of asthma exacerbations in both children and adults [35, 36]. Uller *et al*. [37] demonstrated an exaggerated immune response to mimicked viral stimulation in primary bronchial epithelial cells obtained from patients with asthma compared with a healthy control group. This dysregulated immune response to viral stimulation was expressed as an overproduction of the alarmin TSLP and a decreased production of the antiviral cytokine type 1 (T1) interferon (IFN)-β [37]. Mechanisms related to oxidative stress, as well as the action of tryptase or chymase (mast cell proteases) on the airway epithelium, may also reduce levels of both T1 and type 3 (T3) IFNs (such as IFN-λ) [38, 39]. Decreased levels of T1 and T3 IFNs have also been reported in the lung in different cohorts of patients with asthma [38, 39]. Of note, in bronchial epithelial cells and in an *in vivo* mouse model of asthma exacerbation*,* house dust mites (HDMs), a common asthma allergen, has been found to directly reduce viral-induced epithelial T1 and T3 IFN levels [40]. More recently, Cerps *et al*. [41] investigated the role of HDM allergen sensitisation and effects of HDM allergen exposure on viral mimic-challenged human bronchial epithelial cells from patients with asthma. In this study, it was demonstrated that HDM allergen exposure of human bronchial epithelial cells resulted in the reduction of IFN-β and β-defensin in response to viral infection [41]. Additionally, antiviral IFN-β expression was also reduced in patients with HDM allergen sensitisation *versus* those not sensitised to HDM allergen [41]. Hence, it has been suggested that sensitisation to HDM allergens is a risk factor for dysregulation of the innate immunity of the lung. Interestingly, Woehlk *et al*. [42] demonstrated that allergen immunotherapy targeting HDM sensitisation enhanced airway epithelial antiviral immunity in patients with allergic asthma.

Activation of the innate immune system of the lung by allergens or viral stimulation also promotes the production of IgE [43]. Upon exposure to certain inhaled allergens and viruses, expression of the high-affinity IgE receptor FcεRI is upregulated in the airway epithelium of patients with asthma [43, 44]. Furthermore, re-exposure to allergens can result in the cross-linking of FcεRI on tissue mast cells, leading to degranulation and subsequent bronchoconstriction [43]. Accordingly, IgE is regarded as a key biomarker in asthma and serum IgE levels have been shown to correlate closely with the severity of asthma [45].

Airway epithelium cell samples can be taken during bronchoscopy and cultured *in vitro* to examine the specific role of the airway epithelium [46]. A study using this methodological approach has shown that both asthma phenotype and severity reflects the airway epithelial immune response to viral stimulation [46]. In this study, an increased production of IL-33 and TSLP was associated with increased asthma severity, whereas increased production of pro-inflammatory cytokines was linked to decreased production of IFN-β [46].

In addition to environmental triggers, a genetic component contributes to disease risk in patients with asthma. Genome-wide association studies have identified novel risk alleles and loci, with many of the asthma susceptibility genes expressed in the airway epithelium [22]. For example, *IL1RL1/IL18R1*, *IL33* and *TSLP* have emerged as important genes associated with the development of asthma [22]. Furthermore, several genes linked to epithelial homeostasis, differentiation or barrier immunity have also been identified, including protocadherin 1, cadherin-related family member 3 and human leukocyte antigen-DQ [47]. Thus, asthma susceptibility genes may play a causal role in epithelial barrier and immune dysregulation in patients with asthma.

Given that epithelial barrier defects are linked with chronicity and severity of airway inflammation, restoring barrier integrity may become a useful approach in the treatment of asthma and other airway diseases [48]. Treatments that specifically target epithelial dysfunction-driven inflammation are currently under investigation [49]. While a number of therapies that target cytokines that act downstream of the epithelium, such as IL-5 and IL-4/IL-13, have demonstrated efficacy in asthma [50], targeting epithelial alarmins, which are broad upstream mediators of the inflammatory response, has become an attractive therapeutic option in asthma management [51]. Indeed, inhibition of IL-33 and its receptor ST2 have both shown preliminary efficacy for treatment of patients with asthma and for patients with COPD [49, 52, 53]. Tezepelumab, which targets TSLP, has demonstrated efficacy in patients with T2-high asthma but also in phenotypes with lower levels of T2 inflammation [54]. In a novel mechanistic study using samples from patients with uncontrolled asthma in the randomised, double-blind, placebo-controlled phase 2 UPSTREAM trial, tezepelumab decreased production of IL-33 in bronchoalveolar lavage at 12 weeks [55]. Moreover, tezepelumab treatment reduced viral stimulation-induced epithelial production of IL-33 in bronchial epithelial cells as well as downstream T2 cytokine levels, while maintaining both T1 and T3 IFN levels [56]. These data indicate that a dysregulated immune response of the airway epithelium, seen particularly in patients with severe asthma, can be targeted by the inhibition of TSLP, which also results in reduced levels of IL-33 and T2 cytokines.

### Mucus overproduction, airway remodelling and airway hyperresponsiveness

Airway remodelling describes abnormal changes that occur in both the epithelium and submucosa of the airway, including goblet cell metaplasia, mucus plugging, airway smooth muscle cell hyperplasia, subepithelial matrix protein deposition and fibrosis, and overexpression of angiogenic factors [57, 58]. The airway epithelium plays an important role in driving airway remodelling in asthma [30]. Under healthy conditions, goblet cells contribute to homeostasis in the mucosal epithelia [59]. However, in patients with asthma, epithelial differentiation trajectories are disrupted, resulting in an increase in goblet cells, with associated hypersecretion of mucins and a decrease in mucociliated cells [60, 61]. This subsequently leads to an increase in stored mucins in the epithelium and in secreted mucins in sputum [59]. Airway epithelial cells also produce epidermal growth factor receptor ligands in response to pollutants and mechanical damage, and a subsequent downstream cascade leads to mucus secretion [62]. These processes can lead to the formation of mucus plugs [30].

**FIGURE 3:**
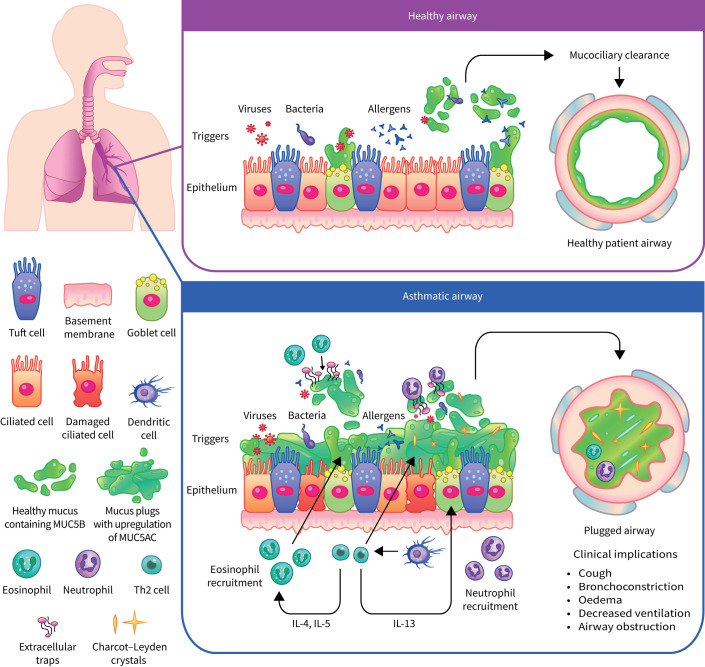
Mucus plugging in healthy and asthmatic airways. When particles enter a healthy airway, they are captured in secreted mucus and expelled by the action of ciliated cells in a process known as mucociliary clearance. When particles enter an asthmatic airway, ineffective mucus clearance occurs and particles remain trapped. Several immune factors that correlate with ineffective mucus clearance have been identified: Th2 cells produce IL-13, which contributes to upregulation of MUC5AC, and IL-4 and IL-5, which contribute to eosinophil recruitment. Eosinophils and neutrophils release extracellular traps to clear pathogens, which increase airway mucus viscosity. Eosinophils are associated with Charcot–Leyden crystal protein formation, which induces mucus secretion. Eosinophils also increase the stimulation of MUC5AC. The formation of mucus plugs that occlude the airway has a number of clinical implications including cough, bronchoconstriction, oedema, decreased ventilation and obstruction of the airway. IL: interleukin; Th: T-helper.

The biology of mucus plugs is not yet fully understood, but it is known that they contribute to a large proportion of airway diseases by both physically obstructing the airways and sustaining the inflammatory response (figure 3) [63]. Several immune factors that correlate with severe mucus plugging have been identified, including sputum eosinophilia and high levels of IL-5 and IL-13 [63]. Advances in semi-quantitative scoring of mucus plugging by computed tomography (CT) have led to an improvement in the assessment of mucus plugging in patients [64]. This research has shown that mucus plugs can reside in the airways for over 3 years and that many patients can have mucus plugs that show resistance to bronchodilators or corticosteroids [63].

Mucus plugs in the lower airway will routinely reform as a consequence of a permanently altered epithelium [64], similar to the nasal polyps observed in patients with chronic rhinosinusitis [65]. A study by the Severe Asthma Research Program has demonstrated that mucus hypersecretion is not a reliable correlate of mucus plugs as identified by CT scans [64]. Moreover, differences in the quality, as well as the quantity of mucus are responsible for the failure to clear inhaled pathogens in asthma. Biophysical properties of the mucus, namely elasticity and viscosity, increase, resulting in highly tenacious mucus which cannot be easily expectorated [66]. This is partly due to an upregulation of MUC5AC, a highly branched mucin which can be crosslinked through disulphide bridges [67, 68]. Patients with T2-high asthma consistently show an increased production of MUC5AC, a process mediated by both IL-13 and epidermal growth factor [69]. Eosinophil–epithelial interactions have been shown to significantly increase the stimulation of MUC5AC secretion [70]. Galectin-10, also known as Charcot–Leyden crystal protein [71], is another eosinophil product with distinct functions in the epithelium. Following eosinophil activation and cell death, galectin-10 can spontaneously auto-crystallise in the extracellular space [72], inducing mucus secretion and IgE production, as demonstrated in a humanised mouse model of asthma [73]. Results from a mouse model have demonstrated that specific antibodies are capable of dissolving crystals within mucus and resolving features of T2 inflammation. Consequently, these crystals represent a novel therapeutic target for reduction of mucus plugs in T2 asthma [73]. Preliminary asthma studies have suggested that, in some patients, treatment with biologics decreases the frequency of mucus plugs observed on CT scans; in a randomised, placebo-controlled trial, treatment with tezepelumab resulted in a reduction in mucus plugging [74]. Furthermore, in observational studies, therapies that target IL-5 have been shown to reduce the numbers of mucus plugs in approximately 60% of patients [75]. The number of mucus plugs was also reduced in approximately 60% of patients after 16 weeks of dupilumab (anti-IL-4/13) treatment [76].

In addition to mucus plugging, increased airway smooth muscle mass and increased subepithelial fibroblasts are the strongest predictors of airflow limitation [77–79]. These remodelling changes result in thickening of the airway wall and luminal constriction that is visible on quantitative CT scans [80]. The epithelium orchestrates these features of airway remodelling through processes including the actions of the alarmins TSLP, IL-33 and IL-25, which activate fibroblasts and airway smooth muscle cells [30, 81–83]. Of note, pre-clinical studies have shown that targeting IL-13 and TSLP leads to an increase in the size of the airway lumen [84–86].

Airway hyperresponsiveness (AHR) results from increased airway smooth muscle contraction that can occur in response to direct or indirect stimuli [87] and is partly independent of the underlying inflammatory profile [87, 88]. Patients with asthma experience higher levels of mast cell infiltration into the airway smooth muscle than healthy people [89, 90]. Mast cells, once activated through the T2 immune cascade initiated by the epithelium, release mediators such as histamine, prostaglandin D_2_ and cysteinyl leukotrienes, which results in contraction of airway smooth muscle cells [91]. Key cytokines released from mast cells include the T2 cytokines, IL-4 and IL-13 [92]. IL-13 leads to activation of airway smooth muscle cells with altered tone inducing bronchoconstriction [93]. Importantly, IL-33 and TSLP can activate mast cells, leading to cytokine release and promoting airway smooth muscle contraction [94]. In experimental and clinical studies, inhibition of TSLP led to improvements in AHR following allergen, methacholine and mannitol challenges [55, 86, 95].

### The role of the epithelium in chronic rhinosinusitis with or without nasal polyps

Similar to the epithelium of the lower airway, the epithelium of the upper airway is directly involved in antimicrobial defence and initiates inflammatory responses to various external triggers (figure 2) [96, 97]. Indeed, impairment of epithelial barrier function has been documented in a number of airway inflammatory diseases including chronic rhinosinusitis, allergic rhinitis and asthma [48]. In line with this, the structural and immune responses of the upper and lower airway are regarded to be similar and often patients can present with co-occurrence of upper and lower airway inflammatory diseases [98].

Epithelial dysfunction occurs in patients with both allergic and nonallergic chronic rhinopathy and in patients with chronic rhinosinusitis with or without nasal polyps (CRSwNP and CRSsNP, respectively) [99]. Although the pathogenesis of CRS remains to be elucidated, there is increasing evidence that impairment of the epithelial barrier plays an important role [100]. Of note, the aetiology of CRS involves several factors including epithelial barrier dysfunction, impaired mucociliary clearance, a dysfunctional immune response and excessive tissue remodelling [101]. Similarly to the lower airways, epithelial dysfunction in the upper airways is the result of complex interaction between genetic factors and environmental triggers [22]. Epithelial dysfunction in the upper airways results in the production of the alarmins TSLP and IL-33, which are capable of inducing T2 inflammation, but with a possible concomitant T1 or T3 inflammatory response [102]. Other consequences of epithelial dysfunction in the upper airway include tissue remodelling and an increase in nasal hyperreactivity, whereby reactions of the nasal epithelium to chemical and physical stimuli are exacerbated [103, 104]. Evidence suggests that there is a high expression of alarmins at the tissue level in the epithelium of patients with CRSwNP [105, 106]. The increased expression of alarmins is observed in response to virus-like stimuli, suggesting that viral infections may have a role in the pathogenesis of CRSwNP [107]. Several studies have also reported upregulated expression of IL-33 and its receptor serum stimulation 2 in nasal polyps at both the mRNA and the protein level [108, 109].

Recommended treatments for CRSwNP and CRSsNP include nasal irrigation with a saline solution, alongside topical corticosteroids [110]. OCS are used as an additional short-term treatment to reduce polyp size in patients with CRSwNP, but they can potentially lead to serious, long-term adverse effects [110]. Endoscopic sinus surgery may be required if treatment with OCS is not effective; however, the benefits are often short lived [110, 111]. Reboot surgery is a surgical operation for CRSwNP that eliminates the entire sinus mucosa. It is interesting to note that this procedure is associated with an almost normal re-epithelialisation, with disappearance of the submucosal inflammatory component [112]. Another method of re-epithelialisation is being investigated in patients with COPD; bronchial rheoplasty uses pulsed electric fields to ablate goblet cells in the large airways to reduce excess mucus production and inflammation [113]. Therefore, treatments of airway disease using innovative techniques that have an impact on the epithelium may be emerging; however, the evidence for the efficacy of these treatments is preliminary and additional investigations are required.

## The airway epithelium in context: clinical biomarkers and implications for patient care

### Assessing the epithelium in asthma

T2 airway inflammation is associated with exacerbations, lung function decline and airway remodelling in patients with severe asthma [114]. The secretion of T2 cytokines generates biomarkers that may be clinically utilised to determine the nature of the airway inflammation (*e.g.*, eosinophilic) [50, 114]; these biomarkers include blood eosinophil count, serum total IgE level and *F*_ENO_ level [115, 116]. Testing for changes at the cellular level can also be used as a predictor of asthma; for example, bronchial provocation tests such as methacholine challenge and mannitol inhalation are, respectively, direct and indirect measures of AHR [117]. The results of these tests may be considered functional biomarkers for asthma. However, none of these measures directly evaluate the state of the epithelium. Tests of epithelial barrier integrity and function could potentially improve phenotyping of patients with asthma if they could be performed in routine clinical practice.

IL-13 has long been considered one of the T2 cytokines responsible in large part for airway inflammation, remodelling and AHR [118, 119]. Together with IL-4, IL-13 enhances subepithelial fibrosis, mucus production *via* goblet cell proliferation and collagen deposition [120]. Both IL-13 and IL-4 are thought to induce epithelial barrier dysfunction by enhancing the expression of histone deacetylases 1 and 9, whose activity inversely correlates with epithelial integrity [121]. IL-13 may also be associated with the production of nitric oxide through the induction of nitric oxide synthase in the epithelium [122]. However, IL-13 has also been shown to have positive effects on epithelial integrity. For example, airway epithelial cells synthesise and release IL-13 in response to mechanical injury, promoting the repair of epithelial damage [123]. Furthermore, in a model of epithelial injury, IL-13 stimulated the production and release of heparin-binding epidermal growth factor and transforming growth factor-α which was found to promote wound closure [124]. Moreover, IL-13 signalling through the IL-13Rα2 receptor is responsible for stimulating wound repair [125].

Overall, these and other experiments demonstrate that IL-13 signalling is essential to airway respiratory health, with imbalances in receptor density or signalling of IL-13 potentially leading to impaired epithelial repair, chronic inflammation and airway remodelling [125, 126]. Given the importance of IL-13 to the state of the epithelium, serum IL-13 level could potentially be considered a biomarker of epithelial health if it could be assessed in routine clinical practice, although it may lack specificity given its wide-ranging role in asthma pathophysiology. The identification of specific biomarkers of epithelial health could be of value for phenotyping patients with severe asthma should therapies that improve epithelial integrity and function become available.

### Future direction for patient care: how can we move towards altering the disease trajectory in severe asthma?

A new paradigm for asthma management has emerged in recent years. Clinical remission is now being considered as a treatment goal for patients with asthma [124, 127, 128]. Historically, remission has been thought of in terms of spontaneous remission, defined as a person who had no wheeze symptoms in the past 3 years and had not used bronchodilators, OCS or ICS in the same time period [129]. In a long-term cohort study that observed patients with asthma from childhood until the age of 50 years in Australia, up to 50% of individuals experienced spontaneous remission [129]. However, asthma remission is now described as a high level of disease control, including the absence of symptoms and exacerbations and normalisation of lung function, with or without ongoing treatment [127]. When considering how asthma remission might be achieved, it is important to consider the pathophysiology of clinical asthma remission [127]. The mechanisms underlying whether asthma persists or undergoes remission are not yet fully understood but may in part involve the epithelium [130]. There is some evidence that bronchial biopsies show enrichment for genes related to the ciliated epithelium in patients who have achieved asthma remission [130]. Furthermore, when comparing patients in remission with healthy individuals, differences in DNA methylation were observed in genes associated with epithelial cells [130]. The airway epithelium is a dynamic tissue with multiple components [131] and it is not clear whether the bronchial epithelium in patients with remitted asthma returns to a healthy structure [132]. Furthermore, those who achieve clinical asthma remission may still have some level of inflammation, AHR and airway remodelling [132]. Therefore, complete asthma remission also requires normalisation of the underlying pathology (for example, resolution of airway inflammation and epithelial function) in addition to clinical remission [127].

Preliminary studies with biologics have shown some evidence of prolonged disease control in patients with asthma [127]. These observations were the starting point for asthma clinicians and researchers to investigate the potential for disease remission as described in rheumatology, oncology and other chronic inflammatory diseases. Following this, several studies with biologics have shown evidence of clinical remission when it is assessed as a clinical end-point [127, 133, 134]. However, additional studies are needed. It would be of interest to examine whether disease progression can be altered with biologics leading to effective repair of damage to the epithelium and long-term clinical remission.

## Identifying knowledge gaps and the future research direction

**FIGURE 4:**
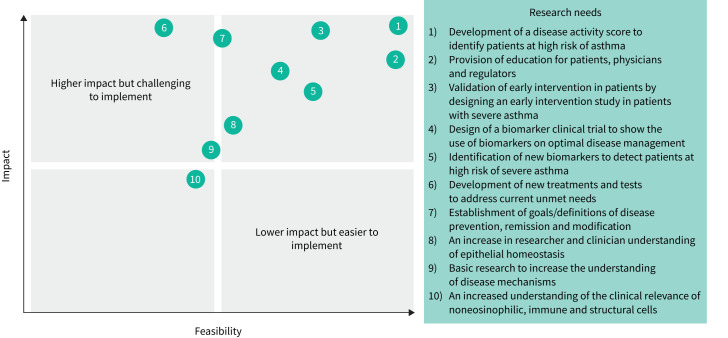
Knowledge gaps in the epithelial era of respiratory science prioritised by research needs. Key knowledge gaps were prioritised in terms of feasibility of implementation and impact of the research. Numbers 1–10 represent the order of prioritisation of research needs.

As we look to the future of asthma management, the authors of this review have identified key areas that require further research to improve our understanding of epithelial science. As part of the Epithelial Science Expert Group meeting, workshops were held to identify and prioritise the knowledge gaps or research needs in epithelial science. The feedback from the workshops was subsequently collated to agree on key themes. These themes were then categorised in a consensus group meeting and the ten most important research needs were identified by the group (figure 4). Here, strategies are suggested to address knowledge gaps, prioritised by feasibility and impact.

Several key focus areas that would have the most impact on addressing the current unmet needs in asthma management have been identified. There is a need to define and support early assessment of asthma to characterise patients at high risk of severe asthma and to avoid disease progression. Patients with asthma are not being diagnosed early enough in the disease lifecycle; patients with severe asthma should be considered as having “too-late” asthma. A “predict and prevent” approach is needed to identify patients with severe asthma as early as possible, to prevent disease progression and to target clinical or complete remission. A recent study has shown evidence of the use of deep learning models to predict patients who are at high risk of asthma or those who are likely to require readmission to hospital following an exacerbation [135]. Use of tools such as this may be of benefit in the future to identify patients with severe asthma as early as possible.

Another key area that requires further research to improve our understanding of epithelial science is an increased awareness of asthma progression. This includes recognising the point at which full repair of the epithelium may not be achievable. Therefore, it would be beneficial to develop a disease activity score to identify patients at high risk of severe asthma and to measure markers of disease progression. For example, a disease activity score has been implemented for the management of other chronic diseases such as rheumatoid arthritis [136]. In addition, the optimal use of existing biomarkers is required to prevent disease progression. Biomarkers that are currently used in clinical practice could be combined with other markers, for example nasal polyps, to identify patients at high risk of disease progression in severe asthma. The routine detection of *F*_ENO_ and sputum eosinophils in clinical practice could also aid clinicians in the diagnosis of patients at high risk of severe asthma, thereby preventing disease progression. One strategy to assess early intervention in asthma would be a clinical study in patients with high *F*_ENO_ levels and a high eosinophil count on at least two occasions. This study would assess the impact of early intervention with biologics on the disease trajectory of patients with severe asthma.

Education for patients, clinicians and regulators was identified as another key area that would impact the current unmet needs in asthma management. This is a limiting factor when influencing patient treatment pathways. In line with this, clear and universal definitions for disease prevention, progression and remission would be helpful to establish defined targets for clinicians. The development of new treatments and diagnostic tests as well as the identification of new biomarkers will also be required to address the current unmet needs. For example, research and identification of new biomarkers, particularly linked to epithelial or smooth muscle cells, may be used to identify patients at high risk of severe asthma. Additionally, identifying biomarkers of epithelial damage or therapeutic strategies aimed at restoring epithelial barrier function will be important to treat patients with severe asthma. Finally, it is necessary to support basic research to increase the understanding of the epithelial science, including the cellular mechanisms that underpin mucus overproduction, AHR, airway remodelling and repair.

## Conclusion

Although asthma research and management has improved over the last few years, several unmet needs remain. It is well established that asthma is a complex disease and several key features of asthma are now known to be driven by the airway epithelium. As we move into the epithelial era, a better understanding of the central role of the epithelium in both upper and lower airway disease is critical. A number of features of the disease need to be considered, including mucus production and airway remodelling. Furthermore, it is important to better understand the events which result in severe asthma and aim for earlier intervention. It is only with optimal use of current biomarkers and the development of new biomarkers that clinicians will be better able to treat severe asthma. An increased understanding of epithelial dysfunction will determine if we can alter disease progression and achieve clinical remission.

**Questions for future research**

- Development of a disease activity score to identify patients at high risk of asthma
- Provision of education for patients, physicians and regulators
- Validation of early intervention in patients by designing an early intervention study in patients with severe asthma
- Design of a biomarker clinical trial to show the use of biomarkers in optimal disease management
- Identification of new biomarkers to detect patients at high risk of severe asthma
- Development of new treatments and tests to address current unmet needs
- Establishment of goals/definitions of disease prevention, remission and modification
- An increase in researcher and clinician understanding of epithelial homeostasis
- Basic research to increase the understanding of disease mechanisms
- An increased understanding of the clinical relevance of non-eosinophilic, immune and structural cells
